# The Efficacy and Safety of Tyrosine Kinase Inhibitors for Von Hippel–Lindau Disease: A Retrospective Study of 32 Patients

**DOI:** 10.3389/fonc.2019.01122

**Published:** 2019-11-01

**Authors:** Kaifang Ma, Baoan Hong, Jingcheng Zhou, Yanqing Gong, Jiangyi Wang, Shengjie Liu, Xiang Peng, Bowen Zhou, Jiufeng Zhang, Haibiao Xie, Kenan Zhang, Lei Li, Desheng Cai, Zixin Wang, Lin Cai, Kan Gong

**Affiliations:** ^1^Department of Urology, Peking University First Hospital, Beijing, China; ^2^Hereditary Kidney Cancer Research Center, Peking University First Hospital, Beijing, China; ^3^Institute of Urology, Peking University, Beijing, China; ^4^National Urological Cancer Center, Beijing, China; ^5^Department of Urology, Beijing Cancer Hospital, Beijing, China; ^6^Beijing Institute for Cancer Research, Beijing, China; ^7^Department of Urology, Fudan University Shanghai Cancer Center, Shanghai, China; ^8^Department of Urology, National Center of Gerontology, Beijing Hospital, Beijing, China; ^9^Department of Urology, The Second Affiliated Hospital of Nanchang University, Nanchang, China

**Keywords:** von Hippel-Lindau disease, tyrosine kinase inhibitors, renal cell carcinoma, efficacy, safety, sunitinib, sorafenib, axitinib

## Abstract

**Background:** Von Hippel-Lindau (VHL) disease is an autosomal-dominant hereditary cancer syndrome. Currently, studies on tyrosine kinase inhibitor (TKI) therapy for VHL disease are scarce. In this study, we retrospectively evaluated the efficacy and safety of four TKIs in patients with VHL disease.

**Methods:** Patients diagnosed with VHL disease who were receiving TKIs were recruited. Patients were treated with sunitinib (*n* = 12), sorafenib (*n* = 11), axitinib (*n* = 6), or pazopanib (*n* = 3). The therapeutic response was evaluated according to the Response Evaluation Criteria in Solid Tumors (RECIST) version 1.1.

**Results:** From July 2009 to September 2018, 32 patients with VHL disease were eligible and included in this study. The median duration of TKI therapy was 22 months (IQR 8.5–44.75), and the median follow-up period was 31.5 months (IQR 13.5–63.5). According to the RECIST, 9 (28%) of 32 patients showed a partial response, 15 (47%) achieved stable disease, and eight exhibited continued disease progression. A partial response was observed in 11 (31%) of 36 renal cell carcinomas, 4 (27%) of 15 pancreatic lesions, and 1 (20%) of five central nervous system (CNS) hemangioblastomas. The average tumor size decreased significantly for renal cell carcinomas (*P* = 0.0001), renal cysts (*P* = 0.027), and pancreatic lesions (*P* = 0.003) after TKI therapy. Common side effects included hand–foot skin reactions, diarrhea, alopecia, thrombocytopenia, and fatigue.

**Conclusions:** Partial alleviation of VHL disease-related tumors can be achieved by TKI therapies in some patients, providing an alternative treatment strategy, and the side effects of TKIs are acceptable. Larger prospective studies are warranted to further evaluate the efficacy and safety of TKIs in patients with VHL disease.

## Introduction

Von Hippel-Lindau (VHL) disease (OMIM 193300) is an autosomal-dominant, multiorgan, familial neoplastic syndrome that results from a germline mutation in the *VHL* tumor suppressor gene (1–3). The incidence of the *VHL* mutation is ~1 in 36,000 live births, and the penetrance is >90% by 65 years of age (3–6). Clinically, VHL disease is characterized by various types of tumors, including central nervous system (CNS) hemangioblastoma (CHB), retinal angioma (RA), renal cell carcinoma (RCC), pancreatic cystic lesions, pancreatic neuroendocrine tumors (PNETs), pheochromocytoma, endolymphatic sac tumors (ELSTs), and epididymal and broad ligament cystadenoma (3, 6, 7). Previously, the prognosis of VHL disease was discouraging, and the median lifespan of patients was reported to be 49 years (8). The most common causes of death were associated with RCCs and CNS hemangioblastomas (8, 9). However, recent studies have reported that the life expectancy of patients with VHL disease has been extended to 64 years (9–11). This improved prognosis may be attributed to several efforts, including earlier diagnosis, active surveillance, and improved treatment of these patients.

In VHL disease, *VHL* mutations lead to the accumulation of hypoxia-inducible factors (HIFs), which activate multiple downstream genes, such as vascular endothelial growth factor (VEGF), erythropoietin, platelet-derived growth factor β (PDGF-β), and transforming growth factor α (TGF-α) (12, 13). Currently, small-molecule tyrosine kinase inhibitors (TKIs), including sunitinib, sorafenib, axitinib, and pazopanib, mainly target the VEGF pathway by inhibiting VEGF ligands or its receptors (14–16). Several studies have reported clinical outcomes in patients with VHL disease treated with TKIs (17–21). A pilot trial by Jonasch et al. (17) assessed the activity and safety of sunitinib in 15 patients with VHL disease, and their results revealed that 6 of the 18 RCCs (vs. none of the CHBs) exhibited a partial response, while the sunitinib dose had to be reduced in 10 patients (17). Only one report has described sorafenib treatment in patients with VHL disease, the results of which showed that low-dose and long-term sorafenib treatment may be an effective option for patients with recurrent RCC (22). Recently, Jonasch et al. completed a prospective study of pazopanib in patients with VHL disease, which revealed that 13 of 31 patients (42%) achieved an objective response and that responses were observed in 31 (52%) of 59 RCCs (20). However, there are currently no studies examining axitinib treatment in patients with VHL disease.

Previous studies have demonstrated that TKIs administered for VHL disease-related tumors may be partially effective and tolerable in most cases. However, the clinical effects of different types of TKIs on various types of tumors in patients with VHL disease are still insufficiently investigated. Thus, in this study, we retrospectively summarized the efficacy and side effects of TKIs for the treatment of patients with VHL disease in a single center. The results showed that TKIs are effective, have acceptable side effects, and are a favorable option for these patients.

## Materials and Methods

### Medical Ethics

This study was approved by the Medical Ethics Committee of Peking University First Hospital (Beijing, China). Informed consent was obtained from patients or their legal guardians.

### Patient Recruitment and Assessment

From July 2009 to September 2018, 32 patients with VHL disease (18 males and 14 females) received TKI therapy at Peking University First Hospital. Molecular diagnosis of VHL disease was also conducted in this hospital. The germline *VHL* mutation was identified in 26 of the 32 patients; six patients were diagnosed with VHL disease because the clinical manifestations fulfilled the clinical diagnostic criteria of VHL disease and first-degree relatives carried a germline *VHL* mutation. Therefore, the genotype of *VHL* mutations could be predicted in all patients (Table 1). In this retrospective study, patients with VHL disease were selected non-randomly and mostly included advanced patients (such as those with more metastatic lesions or higher tumor grades), patients who were excluded from partial renal surgery (e.g., bilateral multiple tumors, large tumors, or tumors in proximity to large blood vessels), and patients who had received adjuvant TKI therapy after surgery.

**Table 1:** Demographic characteristics, types of VHL lesions, and VHL mutations in the 32 VHL disease patients treated with TKI therapy.

**Characteristic**	***n* (%)**
Sex	
Male	18 (56)
Female	14 (44)
Mean age of diagnosis RCC (years)	38.7 ± 10.8 (range 21–59)
Mean age of TKI therapy initiated (years)	41.5 ± 11.2 (range 21–66)
Median duration of TKI therapy (months)	22 (IQR 8.5–44.75)
Median follow-up period (months)	31.5 (IQR 13.5–63.5)
**Clinical manifestation**	***n*** **(%)**
Renal cell carcinoma	31 (97)
Renal cyst	22 (69)
Pancreatic tumor or cyst	27 (84)
Pheochromocytoma	6 (19)
Hemangioblastoma	22 (69)
Retinal hemangioma	7 (22)
Endolymphatic sac tumor	1 (3)
Epididymal cystadenoma	4 (21)
**VHL mutation**	**No. of patients with mutation**
p.R167W	3
p.C162W	2
p.W117G	1
p.N90I	2
p.S88R	1
p.S80I	1
p.S65L	2
p.S65P	1
p.R161^*^	2
Small indel	8
Large deletion	9

RCC, renal cell carcinoma; TKI, tyrosine kinase inhibitor; VHL, von Hippel-Lindau;

* *termination codon*.

### Examination of VHL Mutation

Genomic DNA was extracted from peripheral blood of suspected individuals using QIAamp DNA Blood Mini Kit (QIAGEN, Germany) according to instructions. Three coding exons and flanking intronic regions were amplified by polymerase chain reaction using primers as described in our previous publication (23, 24). Direct sequencing was performed to detect missense mutations, splicing mutations, and small indels. Large deletions and duplications were detected by multiplex ligation-dependent probe amplification (MLPA, P016-C2 kit, MRC-Holland, Amsterdam, the Netherlands). All large exon deletions in this study were verified by real-time PCR with primers described by Ebenazer et al. (25).

### Drug Dosage

For sunitinib, a dosage of 50 mg/day was given orally for 28 days, followed by a 14-day break per cycle for several cycles. For sorafenib, a dosage of 800 mg/day divided into two doses was administered orally. For axitinib, a dosage of 10 mg/day divided into two doses was administered orally. For pazopanib, a dosage of 800 mg/day was administered orally.

### Efficacy and Safety Evaluations

We compared changes in the size of VHL disease-associated tumors before and after TKI therapy in the 32 patients with VHL disease. Baseline and follow-up evaluations of the target lesions were conducted using CT or MRI scans. More than 90% of patients were monitored to assess tumor changes by CT/MRI every 3 months so that complete clinical data could be obtained. The Response Evaluation Criteria in Solid Tumors (RECIST, version 1.1) was used to evaluate the therapeutic response. Side effects related to the four TKIs were evaluated using the Common Terminology Criteria for Adverse Events (CTCAE, version 4.0).

### Statistical Analysis

Summary statistics, including the mean, SD, IQR, and median, were used to describe patient characteristics. Kaplan–Meier plots and the log-rank test were used for survival analysis. Comparisons of tumor size before and after TKI therapy were performed with paired-sample *t*-tests using SPSS software (version 22.0, IBM-SPSS, Chicago, IL). SAS software (version 9.4) was used to construct the Swimmer plots, which reflected the patients' therapeutic responses to TKI therapy at 3-month intervals. A *P* < 0.05 was considered to indicate a statistically significant difference.

## Results

**Figure 1:**
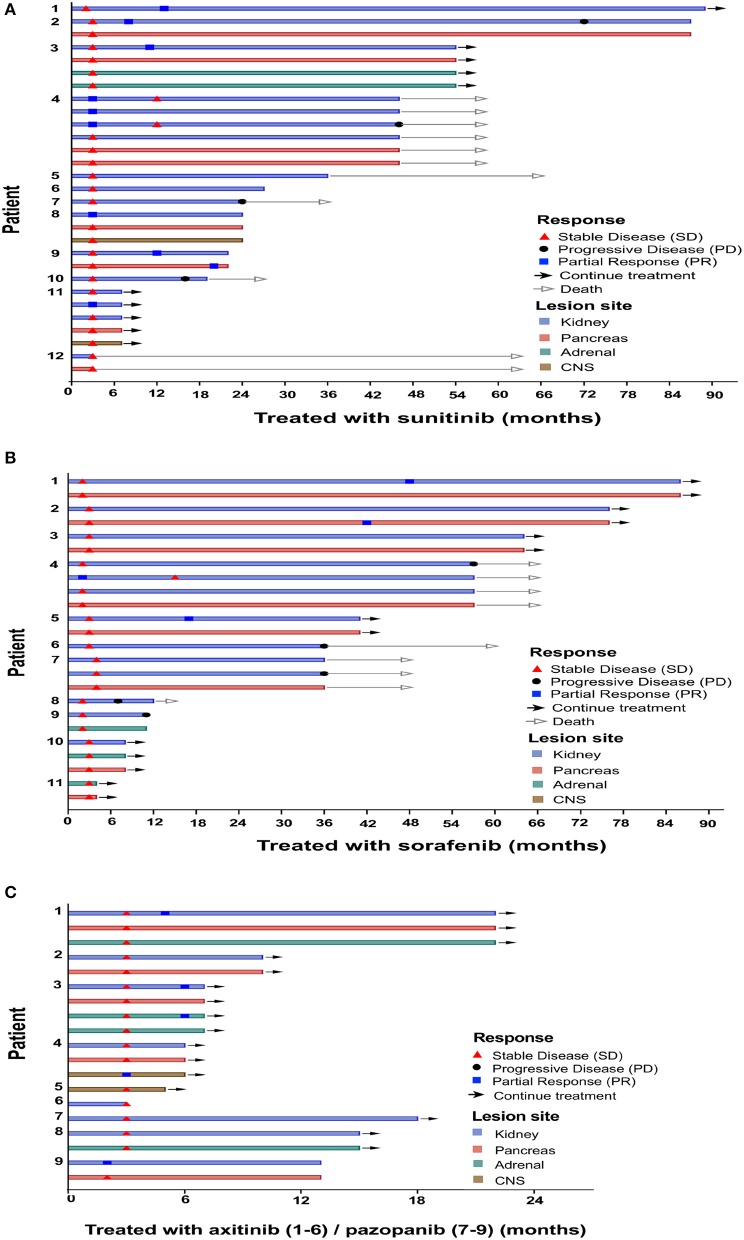
Time point of response appeared. Clinical characteristics per individual. Patients were treated with sunitinib **(A)**, sorafenib **(B)**, axitinib (**C**, patients 1–6), and pazopanib (**C**, patients 7–9).

Demographic characteristics, clinical manifestations, and *VHL* mutations of the 32 patients with VHL disease who received TKI therapy are summarized in Table 1. The mean age at initiation of TKI therapy was 41.5 ± 11.2 years (range 21–66 years), the median period of TKI therapy was 22 months (IQR 8.5–44.75 months), and the median follow-up period was 31.5 months (IQR 13.5–63.5 months). The most common clinical manifestations of these 32 patients were RCC, pancreatic tumor or cyst, and CNS hemangioblastoma. Truncating mutations were present in 19 patients, and missense mutations were present in 13 patients. In this retrospective study, five patients received more than 5 years of TKI therapy, seven patients received 3–5 years of TKI therapy, 11 patients received 1–3 years of TKI therapy, and nine patients received <1 year of TKI therapy (Figure 1).

Overall, after TKI therapy, 9 (28%) of the 32 patients exhibited a partial response, 15 (47%) exhibited stable disease as the best response, and the remaining eight exhibited progressive disease (Figure 1). Of the 12 sunitinib-treated patients, 4 (33%) showed a partial response to the therapy, 4 (33%) achieved stable disease, and 4 (33%) exhibited disease progression. Of the 11 sorafenib-treated patients, 3 (27%) showed a partial response to the therapy, 4 (36%) achieved stable disease, and 4 (36%) exhibited disease progression. Of the six axitinib-treated patients, 2 (33%) showed a partial response to the therapy, and the remaining four patients achieved stable disease. All three pazopanib-treated patients achieved stable disease.

The best responses of lesions after TKI therapy as evaluated by the RECIST are shown in Table 2. Complete response was not found for any of the lesions. The rate of partial response ranged between 17% (1/6 pheochromocytomas) and 31% (11/36 RCCs). Most lesions were categorized as stable disease; the rate of stable disease ranged between 47% (17/36 RCCs) and 83% (5/6 pheochromocytomas). Progressive disease was not observed in patients with renal cysts, pancreatic tumors or cysts, pheochromocytomas, or CHBs, but it was found in 22% (8/36) of RCCs. The best responses of the different lesions after treatment with the four TKIs are summarized in Table 2. Six (40%) of the 15 RCCs presented a partial response, 5 (33%) RCCs were stable, and 4 (27%) RCCs progressed in sunitinib-treated patients; 3 (25%) of the 12 RCCs showed a partial response, 5 RCCs (42%) were stable, and 4 (33%) RCCs progressed in sorafenib-treated patients; 2 (33%) of the six RCCs showed a partial response, and 4 (67%) RCCs were stable in axitinib-treated patients; and all three RCCs were stable in pazopanib-treated patients (Table 2). However, the statistical significance of the responses of RCCs to the four TKIs could not be determined due to an insufficient number of cases.

**Table 2:** Best response of the lesions by RECIST after treatment of one TKI.

**TKI treatment**	**Clinical characteristic**	**Evaluable lesions (*n*)**	**Best response**, ***n*** **(%)**		
			**Partial responsive**	**Stable**	**Progressive**
Total (*n* = 32)	Renal cell carcinoma	36	11 (31)	17 (47)	8 (22)
	Renal cyst	13	3 (23)	10 (77)	0
	Pancreatic tumor or cyst	15	4 (27)	11 (73)	0
	Pheochromocytoma	6	1 (17)	5 (83)	0
	CNS hemangioblastoma	5	1 (20)	4 (80)	0
Sunitinib (*n* = 12)	Renal cell carcinoma	15	6 (40)	5 (33)	4 (27)
	Renal cyst	4	1 (25)	3 (75)	0
	Pancreatic tumor or cyst	5	1 (20)	4 (80)	0
	CNS hemangioblastoma	2	0	2 (100)	0
	Pheochromocytoma	2	0	2 (100)	0
Sorafenib (*n* = 11)	Renal cell carcinoma	12	3 (25)	5 (42)	4 (33)
	Renal cyst	2	0	2 (100)	0
	Pancreatic tumor or cyst	5	2 (40)	3 (60)	0
	CNS hemangioblastoma	1	0	1 (100)	0
Axitinib (*n* = 6)	Renal cell carcinoma	6	2 (33)	4 (67)	0
	Renal cyst	5	1 (20)	4 (80)	0
	Pancreatic tumor or cyst	4	0	4 (100)	0
	CNS hemangioblastoma	2	1 (50)	1 (50)	0
	Pheochromocytoma	3	1 (33)	2 (67)	0
Pazopanib (*n* = 3)	Renal cell carcinoma	3	0	3 (100)	0
	Renal cyst	2	1 (50)	1 (50)	0
	Pancreatic tumor or cyst	1	1 (100)	0	0
	Pheochromocytoma	1	0	1 (100)	0

*TKI, tyrosine kinase inhibitor; n, number of patients who participated in this treatment group; Total represents the response of all patients' lesions to TKIs*.

**Figure 2:**
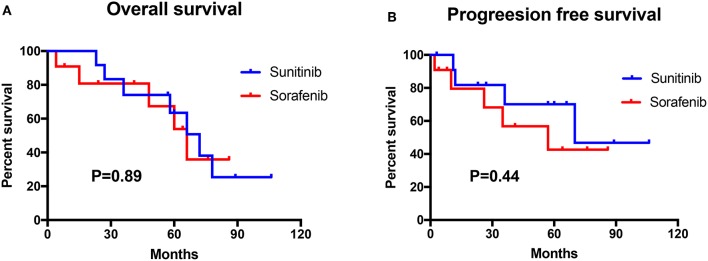
Survival analysis of the sunitinib-treated group (*n* = 12) and the sorafenib-treated group (*n* = 11). **(A)** The overall survival (OS) between sunitinib and sorafenib treatment group. **(B)** The progression-free survival (PFS) between the sunitinib and the sorafenib treatment group.

The mean change in the size of the lesions after TKI therapy is summarized in Table 3. Specifically, the mean change in size was −19.26, −15.92, −18.46, −28.26, and −18.32% for RCCs, renal cysts, pancreatic tumors or cysts, CHBs, and pheochromocytomas, respectively. The changes were statistically significant, except for the change in CHBs, for which the *P*-value was >0.05. In this study, 12 patients were treated with sunitinib, and 11 patients were treated with sorafenib. Nine patients died during follow-up, mainly because of RCC with lung and/or bone metastasis, and eight patients exhibited disease progression during TKI therapy. The median overall survival duration was 72 months for sunitinib-treated patients and 66 months for sorafenib-treated patients, and the median progression-free survival duration was 70 months for sunitinib-treated patients and 57 months in sorafenib-treated patients. Log-rank test results showed that overall survival (*P* = 0.89) and progression-free survival (*P* = 0.44) were not significantly different between the sunitinib- and sorafenib-treated patients (Figure 2).

**Table 3:** Mean change in size compared to the baseline after TKI therapy.

**Tumor type**	**Mean size (standard error)**			***P-*value**
	**Baseline (cm)**	**After therapy (cm)**	**Percent change**	
Renal cell carcinoma	3.79 (0.42)	3.06 (0.42)	−19.26	0.0001
Renal cyst	2.45 (0.39)	2.06 (0.34)	−15.92	0.027
Pancreatic tumor or cyst	2.6 (0.34)	2.12 (0.26)	−18.46	0.003
Hemangioblastoma	2.3 (0.59)	1.65 (0.40)	−28.26	0.073
Pheochromocytoma	3.82 (1.5)	3.25 (1.3)	−14.92	0.056

We observed that nine patients achieved a favorable response, i.e., partial response, and eight patients showed progressive disease after TKI therapy. Comparisons of the clinical characteristics between the patients with a partial response and those with progressive disease are shown in Table 4. Type 1 VHL disease was found in all of the patients (8/8) in the progressive disease group and in 44.4% of the patients (4/9) in the partial response group (Table 4). Missense mutations were found in more than half (5/9) of the patients in the partial response group and in 25% (2/8) of the patients in the progressive disease group. In addition, the mean RCC diameter before TKI treatment was 3.2 cm in the partial response group, which was less than the mean diameter of 5.9 cm in the progressive disease group.

**Table 4:** Characteristics of patients between the PR group and the PD group.

**Characteristic**	**Partial response group**	**Progressive disease group**
No. of patients	9	8
**Sex**		
Male	4	5
Female	5	3
**Family history**		
Yes	7	7
No	2	1
**Mutation Type**		
Missense	5	2
Truncating	4	6
**Clinical type**		
Type 1	4	8
Type 2	5	0
**Renal cell carcinoma**		
Unilateral	2	0
Bilateral	7	8
**Metastasis**		
Yes	0	8
No	9	0
**Survival**		
Yes	8	1
No	1*	7
Mean RCC diameter before TKI therapy (cm)	3.2	5.9
Mean RCC diameter after TKI therapy (cm)	2	4.8
Mean age of RCC diagnosis (years)	35.7	41.1
Mean age of TKI treatment initiation (years)	36.3	46.5
Mean duration of TKI therapy (months)	44.9	37.3
Mean follow-up period (months)	56.3	49.6

PR, partial response; PD, progressive disease; RCC, renal cell carcinoma, ^*^patient who died after brain stem surgery.

The most common side effects were hand–foot skin reactions, diarrhea, alopecia, hypertension, thrombocytopenia, and back pain in patients treated with one of the four TKIs (Table 5). These side effects were slight or mild in most patients, and severe side effects (grade 5) were not recorded. The prevalence of hand–foot skin reactions, diarrhea, and fatigue in sunitinib-treated patients was similar to that in previous studies of the side effects of sunitinib. The most common side effects were hand–foot skin reactions, diarrhea, alopecia, and thrombocytopenia in sorafenib-treated patients and hypertension, hand–foot skin reactions, and back pain in axitinib-treated patients. Other relatively rare side effects included periapical abscess (one case), perforation of the nasal septum due to repeat epistaxis (one case), severe hyperbilirubinemia (one case), hypertensive encephalorrhagia (one case with bilateral pheochromocytomas), and hypothyroidism (six cases). Most of these effects occurred in sunitinib-treated patients. Dose reduction was required in 13 (40.6%) of 32 patients, mainly in the sunitinib- and sorafenib-treated patients. The dose of sunitinib was reduced to 25–37.5 mg/day in six patients, and that of sorafenib was reduced to 400 mg/day in five patients. Dose reductions were rarely necessary in the axitinib- and pazopanib-treated patients.

**Table 5:** Treatment-emergent toxic effects.

**Toxicity**	**All grades**	**Grade 1**	**Grade 2**	**Grade 3**	**Grade 4**	**Grade 5**	**Sunitinib group**	**Sorafenib group**	**Axitinib group**	**Pazopanib group**
Hand–foot skin reaction	20	9	8	3	0	0	7	8	3	2
Diarrhea	19	11	6	2	0	0	8	6	2	3
Alopecia	13	11	2	0	0	0	4	7	1	1
Hypertension	12	5	3	4	0	0	4	3	4	1
Thrombocytopenia	10	7	3	0	0	0	4	5	1	0
Pain	10	5	3	2	0	0	3	2	3	2
Fatigue	8	4	3	1	0	0	6	1	1	0
Hyperbilirubinemia	7	4	2	0	1	0	4	3	0	0
Hypothyroidism	6	3	3	0	0	0	5	0	1	0
Dysgeusia	5	3	2	0	0	0	4	0	0	1
Transaminitis	5	3	2	0	0	0	0	4	0	1
Mucositis	4	3	1	0	0	0	3	0	0	1
Rash	4	3	1	0	0	0	1	3	0	0
Anorexia	4	2	2	0	0	0	4	0	0	0
Nausea	4	2	2	0	0	0	2	1	0	1
Anemia	3	3	0	0	0	0	0	2	0	1
Menolipsis	3	3	0	0	0	0	2	0	1	0
Vomiting	3	2	1	0	0	0	0	2	1	0
Headache	3	2	1	0	0	0	2	0	1	0
Hyperuricemia	2	2	0	0	0	0	1	1	0	0
Infection	2	2	0	0	0	0	2	0	0	0
Dyspnea	1	1	0	0	0	0	0	1	0	0
Edema (head/neck)	1	1	0	0	0	0	1	0	0	0
Elevated creatinine	1	1	0	0	0	0	0	0	0	1
Hydropericardium	1	1	0	0	0	0	1	0	0	0
Epistaxis	1	0	0	1	0	0	1	0	0	0
Encephalorrhagia	1	0	0	0	1	0	1	0	0	0

## Discussion

This retrospective study on TKI therapy for patients with VHL disease in a single center showed that TKIs lessened the disease burden and that the side effects were acceptable. Primary data indicated a clinical benefit for patients with VHL disease with RCCs, pancreatic lesions, pheochromocytomas, renal cysts, and, possibly, CHBs (Table 2). The median duration of follow-up in this study was 31.5 months, which was longer than that in previous reports (17, 20). The longer follow-up period enabled us to observe the survival and TKI resistance of the patients. This is the largest retrospective study of TKIs in patients with VHL disease.

In this study, 32 patients were included, of whom 13 patients' mutation types were missense mutations, and the remaining were truncating mutations. Furthermore, we compared the landscape of VHL mutations with our previous study by Wang et al. and the international research from the Netherlands by Morgan Nordstrom-O'Brien et al. (Supplementary Table 1). We found that the landscape of VHL mutations was similar with previous reports (9, 26). Our study showed that many patients chose to continue TKI therapy because of the benefits of TKIs or the difficulties of surgical resection. Eleven (31%) of the 36 RCCs achieved partial response, 17 (47%) of 36 RCCs achieved stable disease, and 8 (22%) RCCs exhibited progressive disease (Table 2). Therapeutic benefits were also observed in pancreatic lesions and renal cysts. In 12 patients receiving TKI therapy for more than 3 years, we observed that pancreatic lesions and renal cysts were not cancerous and generally did not require treatment. Of the five CHBs, one achieved a partial response after 2 months of axitinib therapy, and of the six pheochromocytomas, 1 (17%) achieved a partial response after 6 months of axitinib therapy, which has rarely been reported in previous studies (21, 27).

Several pilot and retrospective studies have reported that sunitinib may be effective in patients with VHL disease, which is consistent with the results of our study. A phase 2 trial by Jonasch et al. reported that 6 (33%) of 18 RCCs showed partial response and that 19 (91%) of 21 HBs and all of the RAs and PNETs showed stable disease after 6 months of sunitinib treatment in 15 patients with VHL disease (17); common side effects included fatigue, diarrhea, anemia, and hand–foot skin reactions. In 2012, Ali et al. found that sunitinib was effective for PNET in patients with VHL disease (28). In addition, Kim et al. reported that metastatic RCCs treated with sunitinib exhibited a partial response that lasted for a long period of time in four patients with VHL disease (18). In our 12 patients with VHL disease treated with sunitinib, 6 (40%) of 15 RCCs and 1 (20%) of five pancreatic lesions showed a partial response (Table 2); common adverse reactions included hand–foot skin reactions, diarrhea, fatigue, and hypothyroidism.

Only one case report of sorafenib treatment for patients with VHL disease can be found in the literature. Choi et al. reported that low-dose sorafenib maintenance was an effective long-term treatment option for RCCs in patients with VHL disease who needed maximal preservation of renal function (22). We studied the effect of sorafenib in 11 patients with VHL disease, which is the largest retrospective study on sorafenib treatment for patients with VHL disease to date; 3 (25%) of 12 RCCs and 2 (40%) of five pancreatic lesions exhibited a partial response (Table 2), and the common adverse effects included hand–foot skin reactions, alopecia, diarrhea, and thrombocytopenia.

No report has examined axitinib treatment for patients with VHL disease. We assessed the effects and safety of axitinib in six patients with VHL disease and found that 2 (33%) of 6 RCCs, 1 of 3 pheochromocytomas, and 1 of 2 CHBs exhibited a partial response. Side effects mainly included hypertension, back pain, hand–foot skin reactions, and diarrhea. The sizes of CHBs were reduced after axitinib therapy in two patients. One CHB in the right cerebellum decreased from 2.3 × 1.7 cm to 1.9 × 1.4 cm on MRI, accompanied by significant alleviation of hydrocephalus and headache after 3 months of axitinib therapy, and another CHB in the L2–L3 spinal cord exhibited a partial response, with a decrease in size from 3.7 × 1.2 × 1.1 cm to 2.4 × 0.9 × 0.9 cm on MRI after axitinib treatment for 2 months. Therefore, axitinib may be effective for CHBs in patients with VHL disease. Larger prospective studies are warranted to further evaluate the efficacy and safety of axitinib in VHL-related CHBs.

Recently, a prospective study of pazopanib in patients with VHL disease by Jonasch et al. revealed that 13 (42%) of 31 patients achieved objective responses, and lesion site responses were observed in 31 (52%) of 59 RCCs, 9 (53%) of 17 pancreatic lesions, and 2 (4%) of 49 CHBs (20); side effects mainly included fatigue, diarrhea, transaminitis, and skin hyperpigmentation. These researchers suggested that pazopanib is effective for VHL disease and may be a treatment option for these patients. We treated three patients with pazopanib, and one RCC and one pancreatic lesion showed a partial response. Side effects mainly included diarrhea, back pain, and hand–foot skin reactions. Due to the small number of patients, we could not assess the efficacy of pazopanib in patients with VHL disease.

In this study, the renal cyst includes renal simple cyst and renal complex cyst. Although the response of renal cyst of VHL disease to TKIs has not been reported in previous research, we found that some TKIs were used to treat autosomal-dominant polycystic kidney disease (ADPDK) in previous literature; the research considered that TKIs can be used to decrease EGFR tyrosine kinase activity and collecting tubule cyst formation and enlargement in polycystic kidney disease (29, 30). In our study, we found 3 (23%) of 13 renal cyst responses to TKIs. We think that the results may be attributed to renal complex cyst, as the previous article revealed that complex cystic and solid lesions can contain neoplastic tissue that frequently enlarges (3). We think that the neoplastic tissue of renal complex cyst may respond to TKIs therapy, which was consistent with the observations in our clinical practice. Larger prospective studies should be performed to further evaluate the efficacy of TKIs in VHL-related renal cyst.

The reason why organ-specific VHL-derived tumors respond differently to TKIs is unclear until now. We know that VHL disease is a multi-organ cancer syndrome, which is characterized by the development of several benign or malignant tumors and cysts in many organ systems. A previous study by Kluger HM et al. showed that VEGF and VEGF receptors were tightly co-expressed in human RCCs specimen (*P* < 0.001) (31). In 2011, a phase 2 trial by Jonasch et al. found that VEGFR2, pVEGFR2, and phosphorylated-to-total VEGFR2 ratios were statistically significantly higher in the RCC than in the HB samples (*P* = 0.001), and their study reported that 6 of 18 RCCs (33%) responded partially to sunitinib, vs. none of 21 HBs (*P* = 0.014) (17). In our study, RCC shows a higher partial response rate for TKIs compared to other VHL-related tumors. We know that sunitinib, sorafenib, axitinib, and pazopanib are small-molecule inhibitors of vascular endothelial growth factor receptors (VEGFRs) (16, 17, 32). Therefore, we think that tumor-specific genetic lesions or tissue-specific endothelial heterogeneity may explain these differences in response. Future studies will be conducted to further explain the reason why organ-specific VHL-derived tumors respond differently to TKIs.

During TKI therapy, a periapical abscess occurred in one case during the sixth cycle of sunitinib therapy, and the drug was discontinued for 17 weeks to treat the periapical abscess. This patient also presented hypothyroidism in the ninth cycle of therapy, and Euthyrox was administered once per day. Hypothyroidism and perforation of the nasal septum due to repeated epistaxis were observed in one patient. In this study, hypothyroidism was observed in six patients, of whom four were administered Euthyrox during TKI therapy. Among the patients with hypothyroidism, 5 (41.7%) cases occurred in sunitinib-treated patients, which is consistent with the results of previous studies (33). Previous prospective studies indicate that sunitinib can induce hypothyroidism in 36–71% of patients. Hyperbilirubinemia was observed in one patient, who required hospitalization after 15 days of sunitinib therapy. One patient with bilateral giant pheochromocytomas (L: 7.9 × 7.4 cm; R: 8.6 × 4.7 cm) was recommended to undergo TKI treatment primarily because of the risks during surgical resection. Hypertensive encephalorrhagia occurred in one patient after drug discontinuance during the 52nd treatment cycle, and sunitinib treatment was resumed after partial recovery from the cerebral hemorrhage sequelae.

This was a retrospective study, and thus, information bias may exist. In addition, only six patients were treated with axitinib, and three patients were treated with pazopanib, which limited our evaluation of the value of axitinib and pazopanib for patients with VHL disease.

In conclusion, this research is the largest retrospective study of TKIs in patients with VHL disease. Our results showed that TKIs were partially effective for RCCs, pancreatic lesions, and pheochromocytomas, and possibly effective for CHBs, and the side effects were acceptable. Further evaluation of TKIs in patients with VHL disease in larger prospective studies is warranted.

## Data Availability Statement

This manuscript contains previously unpublished data. The name of the repository and accession number(s) are not available.

## Ethics Statement

This study was approved by the Medical Ethics Committee of Peking University First Hospital (Beijing, China). Informed consent was obtained from patients or their legal guardians.

## Author Contributions

KG and LC developed the hypothesis and secured funding. KM wrote the first draft of the paper. JW, SL, XP, BZ, and JZha carried out statistical analyses. JZho, BH, HX, KZ, and LL dealt with figures and tables. YG, DC, and ZW performed data collection. BH revised the manuscript. All authors critically commented on and approved the final submitted version of the paper.

### Conflict of Interest

The authors declare that the research was conducted in the absence of any commercial or financial relationships that could be construed as a potential conflict of interest.
