# Supplementary material for: The Efficacy and Safety of Tyrosine Kinase Inhibitors for Von Hippel–Lindau Disease: A Retrospective Study of 32 Patients
Source: Front Oncol. 2019 Nov 1;9:1122. doi: 10.3389/fonc.2019.01122 (PMC6839035; doi:10.3389/fonc.2019.01122)
Supplement: Supplementary file 1 [file Table_1.DOCX]

**Supplementary Table 1:** The landscape of VHL mutations in this study compared with other studies.

| Mutation type | This study  (N=32 ) | | Our previous study  (N=340, PMID:29330336) | | International study (N=804, PMID:20151405) | |
| --- | --- | --- | --- | --- | --- | --- |
|  | n | percent | n | percent | n | percent |
| Missense | 13 | 40.6% | 165 | 48.5% | 491 | 61% |
| Frameshift | 7 | 21.9% | 175 (Truncating mutations) | 51.5% | 126 | 15.7% |
| Nonsense | 2 | 6.3% |  |  | 106 | 13.2% |
| InF Del/Ins | 1 | 3.1% |  |  | 28 | 6.6% |
| Splice | 0 | 0 |  |  | 53 | 3.5% |
| Large deletion | 9 | 28.1% |  |  | / | / |
| Total | 32 |  | 340 |  | 804 |  |

Note: N, number of patients in this study; n, number of patients in this mutation type; InF Del/Ins, in-frame deletions/insertions; Truncating mutations, all mutation types except missense mutations.
